# Knowledge, Attitude, and Practice of Adult Population towards Blood Donation in Gondar Town, Northwest Ethiopia: A Community Based Cross-Sectional Study

**DOI:** 10.1155/2016/7949862

**Published:** 2016-07-19

**Authors:** Mulugeta Melku, Betelihem Terefe, Fikir Asrie, Bamlaku Enawgaw, Tadele Melak, Yakob Gebregziabher Tsegay, Mohamedamin Areba, Elias Shiferaw

**Affiliations:** ^1^Department of Hematology and Immunohematology, School of Biomedical and Laboratory Sciences, College of Medicine and Health Sciences, University of Gondar, P.O. Box 196, Gondar, Ethiopia; ^2^Department of Clinical Chemistry, School of Biomedical and Laboratory Sciences, College of Medicine and Health Sciences, University of Gondar, P.O. Box 196, Gondar, Ethiopia; ^3^Quality and Safety Office, National Blood Bank Services, P.O. Box 1234, Addis Ababa, Ethiopia; ^4^Department of Medical Laboratory Sciences, School of Biomedical and Laboratory Sciences, College of Medicine and Health sciences, University of Gondar, Gondar, Ethiopia

## Abstract

*Background*. Though World Health Organization recommends 100% voluntary blood donation, the percentage of blood collected from voluntary blood donors and the average annual blood collection rate are extremely low in Ethiopia. The role of adults is crucial to meet the demand of safe blood. Thus, this study aimed to assess knowledge, attitude, and practice of adult population towards blood donation in Gondar town, Northwest Ethiopia.* Method*. A community based cross-sectional study was conducted among 768 adults. Multistage sampling technique together with simple random and systematic random sampling technique was employed. Bivariate and multivariate logistic regression analysis and bivariate correlation analysis were done.* Result*. About 436 (56.8%), 630 (82%), and 141 (18.4%) study participants had adequate knowledge, good attitude, and experience of blood donation, respectively. Secondary and higher educational statuses were significantly associated with adequate knowledge towards blood donation. Participants who were protestant by religion were more likely to have good attitude towards blood donation. Age, self-perceived health status, and religion were significantly associated with blood donation practice.* Conclusion*. Knowledge and attitude towards blood donation are high. However, the level of practice is low. District and national blood banks and transfusion agency should design strategies that promote and motivate the communities to donate blood.

## 1. Introduction

Blood is an invaluable, life-sustaining fluid. Without a sufficient amount of blood, the cells of the human body could not receive adequate oxygen and nutrients they need to survive. Large volume of blood could be lost as a result of numerously varying serious conditions such as road traffic accidents, obstetric and gynecological hemorrhages, surgery, trauma, chemotherapy, and long-term therapies as well as anemia of medical or hematologic conditions or cancer. Because of these blood transfusion is considered as an integral and essential element of a health care system. Besides, blood transfusion is one part of complex medical and surgical interventions which improves the life expectancy and life quality in patients with a variety of acute and chronic conditions. Therefore, blood transfusion is now considered as an indispensable component of medical management of many diseases [1].

Blood donation is philanthropic deed in which the blood of a healthy person had been drawn voluntarily for the purpose of transfusion. The donated blood can be life-saving for individuals who have lost large amounts of blood because of serious accidents, as well as for individuals who have become severely anemic or have very low platelet counts and certain hematological disorders such as leukemia [2]. Besides, children being treated for cancer, premature infants, and children having heart surgery need blood and platelet transfusions to survive [3].

World Health Organization (WHO) recommends countries to focus on young people to achieve 100% nonremunerated voluntary blood donation by 2020. It also recommends that all countries should be self-sufficient in all blood products and that all blood donation should be voluntary, anonymous, and nonremunerated [4]. According to its 2011 report, 107 million blood donations are collected globally; approximately half of these are collected in the high-income countries, home to 15% of the world's population. Blood donation rate in high-income, middle-income, and low-income countries was 39.2, 12.5, and 4.0 donations per 1000 population, respectively. In low-income countries, up to 65% of blood transfusions are given to children under five years of age, whereas, in high-income countries, the most frequently transfused patient group is over 65 years of age, accounting for up to 76% of all transfusions. Compared to the 2004 report, 7.70 million blood donations incensement was noticed from voluntary unpaid donors in 2011. However, majority of countries still collect more than 50% of their blood supply from replacement or paid donors [5].

About 234 million major operations are performed worldwide every year; 63 million people undergo surgery for traumatic injuries, 31 million for treating cancers, and another 10 million for pregnancy-related complications. For all of these procedures, blood transfusion is mandatory [3]. Moreover, the demand of blood for patient management has been growing dramatically due to the sophistication and advancement of clinical medicine. However, the demand and supply have not yet balanced; the demand is escalating. Despite recommendations that all blood donations should be voluntary and nonremunerated, replacement and paid donors are common throughout Sub-Saharan African countries [6]. Surprisingly, 38 African countries collected fewer than 10 donations per 1000 people [5]. There have been gross inadequacy and inequity in access to blood safety in WHO African region [7, 8]. Concurrently, in Sub-Saharan African countries, the need for blood transfusions is high because of maternal morbidity, malnutrition, and a heavy burden of infectious diseases such as malaria [6].

In Ethiopia, there have been gross inadequacy and inequity in access to blood. The national requirement for blood in Ethiopia is between 80,000 and 120,000 units per year, but only 43% is collected [9]. The percentage of blood collected from VBD and the average annual blood collection rate are extremely low. Out of the 44 WHO African countries that reported the percentage of voluntary nonremunerated blood donation (VNRBD), only 22% of blood is being donated by VBD in Ethiopia; the country is classified among countries that have least number of VBD (Group C, countries with <50% VBD) [10].

Adult population are potential source of great interest not only for the blood they could supply but also because of the information on the subject “giving blood” which could promote the spread of healthy lifestyles and acquisition of greater awareness about one's own health and contribute to the development of a mature, responsible, and civic attitude [11]. Voluntary, nonremunerated blood donations are the cornerstone of a safe adequate supply of blood and blood components [12, 13]. Thus, the objective of this research was to assess knowledge, attitude, and practice towards blood donation among adult population in Gondar town, Northwest Ethiopia.

## 2. Materials and Methods

### 2.1. Study Design, Study Period, and Study Population

Community based cross-sectional study was conducted in Gondar town, Northwest Ethiopia, from February to May 2015. The source populations were all adults who were residing in study area at least for 6 months and who were available during data collection period. Those adults who were critically ill and had mental problems were excluded from the study.

### 2.2. Variables

The dependent variables were knowledge, attitude, and practice of blood donation. The independent variables were sociodemographic variables like sex, age, educational status, marital status, religion, and self-perceived health status.

### 2.3. Sample Size Determination

Single population proportion formula, [*n* = (*Zα*/2)^2^ *p*(1 − *p*)/*d* ^2^], was used to calculate the sample size. Due to the lack of published information showing the knowledge, attitude, and practice of blood donation in this particular study area, we took 50% to get the maximum sample size by considering 95% confidence interval, marginal error (*d*) of 5%, and design effect of 2. Then, the final sample size was determined to be 768.

### 2.4. Sampling Techniques

In the first stage of the sampling, three administrative areas (subcities), Lideta, Maraki, and Gebriel, were selected by using simple random sampling technique from the total 12 subcities. In the second stage of sampling, Sanita ketena from Lideta subcity, ketena two from Maraki subcity, and kebele 14 from Gebriel subcity were selected randomly. Then, systematic sampling technique was employed to select households from each of the ketenas/kebeles. The numbers of households sampled from the selected ketenas and kebeles were determined using proportionate-to-population size.

There were a total of 4603 households in three selected kebeles/ketenas: 1800 in Sanita ketena of Lideta subcity, 1960 in ketena two of Maraki subcity, and 843 in kebele 14 of Gebriel subcity. The interval (*K*) value was calculated for each selected kebele/ketena by dividing the total households in each selected kebele/ketena to the corresponding proportional sample size calculated for each ketena/kebele.

The initial household was randomly selected by lottery method. Then other households were selected at every *K*th interval. Whenever more than one eligible adult was found in the same selected household, only one of them was chosen using the lottery method for interview. In the case no eligible candidate was identified in a selected household or the selected household is closed even after revisit, the sampling process continued to the next household in the clockwise direction until getting an eligible person.

### 2.5. Assessment of Knowledge, Attitude, and Practice

Knowledge about blood donation was assessed using 13 general questions which are deemed to be known by general population like place of blood donation, importance of blood donation, and eligibility for blood donation. Each response was scored as “1” for correct response and “0” for incorrect response. The scoring ranges from 13 (largest) to 0 (smallest). Knowledge scores for individuals were calculated and summed up to give the total knowledge score. Participants who correctly responded to more than 50% of knowledge assessing questions were considered as having adequate knowledge about blood donation, whereas those who scored <50% were considered as having inadequate knowledge about blood donation.

Similarly, 14 attitudes related questions were asked, and the responses of each question were scored as “1” for correct response and “0” for incorrect response. The attitude scoring ranges from 14 (largest) to 0 (smallest). Attitude scores for individuals were calculated and summed up to give the total attitude score. Participants who correctly responded to more than 50% of attitude assessing questions were considered as having good attitude towards blood donation, whereas those who scored ≤50% were considered as having poor attitude towards blood donation.

The practice was assessed by asking about history of previous donation and the frequency of donation. The practice was scored from largest (the number of times a donor donated previously) to smallest 0 (never donated before).

### 2.6. Data Analysis and Interpretation

The data were entered using Epi Info version 3.5.1 and then cleaned and analyzed using SPSS version 20 software package. Data cleaning was carried out by running frequency of each categorical variable and cross tabulation of different categorical variables. Descriptive results were summarized as percentage, means, and standard deviations and presented in table. Each of the outcome variables was computed with each independent variable. The association of the independent variable with the categorical outcome variable was measured by calculating odds ratio with *P* value and 95% confidence interval using bivariate and multivariate logistic regression. All independent variables with *P* value less than 0.2 were included in the multivariate models to identify factors associated with knowledge, attitude, and practice towards blood donation. Besides, the relationships between knowledge, attitudes, and practice scores were examined using bivariate correlation analysis. *P* value of less than 0.05 was considered as statistically significant.

### 2.7. Ethical Consideration

The research was conducted after ethical approval letter was given from Research and Ethical Committee of School of Biomedical and Laboratory Science, University of Gondar. In addition, after explaining the importance of study, permission letter was taken from each of the kebeles/ketenas administrators, and an informed consent was obtained from each study participant.

## 3. Result

### 3.1. Sociodemographic Characteristics

From a total of 768 participants, 430 (56%) were male and 338 (44%) were female. More than half of the participants (*n* = 402 (52.3%)) were in the age range of 20–25 years. The median age of the participants was 25 years. About 354 (46.1%) and 189 (24.6%) of the study participants had attained or have been attaining secondary and higher education, respectively (Table 1).

### 3.2. Knowledge of Study Participants

From the total study participants, 436 (56.8%) had adequate knowledge towards blood donation. The mean knowledge score of the participants was 6.62 ± 3.09 SD. Majority (*n* = 704, 91.8%) of the study participants heard the idea of blood donation previously. About 678 (88.3%) study participants thought that the importance of blood donation is to save life, while 24 (3.1%) of them believed that it is to get health assurance (Table 2).

### 3.3. Attitude of the Study Participants

More than three-fourths, 630 (82%), of the respondents had good attitude towards blood donation. The mean attitude score of the participants was 10.30 ± 3.038 SD. Nearly all, 741 (96.5%), of the participants thought that blood donation is important (Table 3).

### 3.4. Practice of Study Participants

Less than one-quarter, 141 (18.4%), of the respondents had an experience of blood donation, while the rest of the participants, 627 (81.6%), never donated blood before. Of those who donated before, 86 (61%) were voluntary donors, while the rest 39% of them were replacement donors. The major reasons mentioned for not donating blood among nondonors were perception of not being fitted to donate blood (21.2%), lack of information on where, when, and how to donate blood (17%), fear of being anemic after blood donation (12.6%), and fear of health risk after donation (12.3%) (Table 4).

### 3.5. Factors Associated with Knowledge

In bivariate logistic regression, age, occupation, marital status, educational status, and self-perceived health status were significantly associated with adequate knowledge about blood donation, while, in multivariate logistic regression controlling confounders, secondary educational status (AOR = 2.28; 95% CI: 1.51, 3.44) and higher educational status (AOR = 2.88; 95% CI: 2.01, 4.12) were significantly associated with adequate knowledge towards blood donation (Table 5).

### 3.6. Factors Associated with Attitude

In bivariate logistic regression marital status, religion, and self-perceiver health status were significantly associated with attitude of the participants, while in multivariate logistic regression religion was the factor which was significantly associated with attitude towards blood donation (Table 6).

### 3.7. Factors Associated with Practice

In bivariate logistic regression analysis, age, sex, religion, marital status, and self-perceived health status were statistically associated with blood donation practice of the respondents, while in multivariate logistic regression analysis, participant's age, sex, religion, and self-perceived health status were found to be significantly associated with practice of blood donation (Table 7).

In addition, we had tried to assess the correlation between knowledge, attitude, and practice scores of the study participants. Knowledge and attitude scores of the participants achieved significant but weak positive correlation (*r* = 0.238; *P* = 0.01). Similarly, knowledge and practice scores of the participants had shown statistically significant positive correlation, even though it is weak (*r* = 0.26; *P* = 0.01). Moreover, the attitude and practice scores of the participants had fair positive correlation (*r* = 0.31; *P* = 0.01).

## 4. Discussion

In this study, an attempt has been made to assess the level and factors associated with knowledge, attitude, and practice of adults on blood donation. From the total study participants, 436 (56.8%) had adequate knowledge regarding blood donation. The result is higher than a study done in Jordan aimed at investigating knowledge and attitude of blood donors and barrier concerning blood donation among 500 blood donors which reported that 28.6% of them had adequate knowledge [14]. The possible reason for this discrepancy might be due to the difference in the sample size.

In this study, more than three-fourths (88.3%) of the participants knew that the importance of blood donation is to save life. The result was higher than a study conducted in Democratic Republic of Congo among 416 participants to assess the knowledge, attitude, and practice of the general population showing that only 183 (44.1%) of them responded that the importance of blood donation is to save life [15]. The difference might be due to variation in sample size and also variation in age of the study participants. In our study, only adult age group, 20–40 years old, were included, whereas in the study of Democratic Republic of Congo participants were in the age range of 18–65 years.

In the current study, multivariate logistic regression showed that educational status was the only variable that significantly associated with the knowledge of participants. Participants who attended or had been attending secondary education (AOR = 2.9; 95% CI: 1.51; 3.44) and higher education (AOR = 2.9; 95% CI: 2.01; 4.12) were more likely to have adequate knowledge towards blood donation. Thus, as the level of education increases, participants' knowledge towards blood donation also increases.

Majority 630 (82%) of the study participants have good attitude towards blood donation. About 282 (36.7%) of them had a perception that blood donation causes anemia. This result is in line with a study conducted in Mekelle City in which 370 (45.9%) of the study participants believed that blood donation causes anemia [16].

In this study, religion was the only variable significantly associated with the attitude of the participants using multivariate logistic regression. Those participants who were catholic and Jewish (AOR = 0.16; 95% CI: 0.05, 0.51) were less likely to have good attitude towards blood donation. Being catholic and Jewish reduces the attitude towards blood donation by 84% compared to being orthodox Christian by religion. This needs further in-depth behavioral study to explore the reason why being catholic and/or Jewish by religion reduces blood donation perception.

In the current study, less than one-fourth, 141 (18.4%), of the study participants had the experience of blood donation. The result is in agreement with studies done in Trinidad and Tobago (18.8%) [17] and north central Nigeria (22.6%) [18]. On the contrary, it is lower than studies conducted in Saudi Arabia (58.2%) [19], Iran (26%) [20], and southern Brazil (32%) [21]. In Saudi Arabia, Iran, and southern Brazil studies, the study participants were in the age of range of 18–50, in the age range of 18–65, and above 20 years of age, respectively. However, in our study, the study's population were within the age range of 20–40 years. Probably, individuals with age above 40 become socially responsible, and they do have increasing tendency to donate blood as supported by Zago et al. [21].

Among donors, 62 (43.9%) donated blood more than once. The rate of previous blood donation is higher than study done in Saudi Arabia (26.4%) [19]. However, it is lower than studies done in Iran (55%) [20]. The result is in contrast to studies done in north central Nigeria [18] and Saudi Arabia [19] in which replacement donors were more frequent than voluntary donors.

More than half, 432 (68.9%), of nondonors stated wrong perception like fear of being anemic, fear of weight loss, fear of health problem, perception of not being fit, and lack of information on where, when, and how to donate blood as a major reason for not donating. This result is consistent with study conducted in Trinidad and Tobago [17].

In current study, multivariate logistic regression showed that participant's age, sex, religion, and self-perceived health status were significantly associated with the practice of participants. Those participants in age ranges of 31–35 years (AOR = 2.61; 95% CI: 1.6; 4.86) and 36–40 years (AOR = 3.8; 95% CI: 2.0; 7.31) were more likely to donate blood as compared to participants in age range of 20–25 years. The possible reason for this might be due to the fact that participants at age range of 30–40 years are in late adulthood stage so that they are assumed to be socially proactive and donate blood. This had also been supported by study done in southern Brazil which revealed that individuals in the age range of 30–49 had higher tendency to be loyal blood donors [21].

In this study, more than half, 94 (66.6%), of donors were male. The result is in line with a study conducted in Togo which showed that majority (61%) of blood donors were male [22]. Males were two times more likely to donate blood compared to females (AOR = 1.7; 95% CI: 1.14; 2.54). This is in agreement with the study done in Yazd, Iran, which reported that significantly higher proportions of men were donors compared to women [23]. The possible reason for this difference with regard to donation practice between women and men might be related with knowledge difference. Culturally, the society is male dominated; and there is disparity in access to education between women and men in Ethiopia [19]. Moreover, our data showed that significant difference was observed in knowledge score between women and men: men had higher score than women (*χ* ^2^ = 22.4; *P* value = 0.049).

In this study, study participants with excellent self-perceived health status were two times more likely to donate blood as compared with those with good self-perceived health status (AOR = 2.23; 95% CI: 1.4; 3.62). This is in line with study done in southern Brazil [21]. People who feel they are healthy are more confident and suitable for donating blood.

In this study, knowledge and attitude scores of the participants had shown significant positive correlation even if the correlation is weak (*r* = 0.238; *P* = 0.01). This indicates that having adequate knowledge leads to having good attitude. Likewise, knowledge and practice scores of the participants had shown positive but weak correlation (*r* = 0.26; *P* = 0.01). This result is in line with a study which was conducted in the city of Yazd to assess the level of knowledge, attitude, and practice regarding blood donation [23]. Meanwhile attitude and practice of the participants have fair (*r* = 0.31; *P* = 0.01) positive correlation. Thus, as the attitude of the participants increases the level of practice also increases.

In the current study, the major reasons mentioned by nondonors for not donating blood were perception of not being fitted to donate blood, lack of information on where, when, and how to donate blood, fear of being anemic after blood donation, and fear of health risk after donation. Even though the extent of the problem varies with race, sociocultural values, and socioeconomic status of the population on which the studies focused, the blood donation barriers reported by nondonors in our study are nearly in agreement with other studies [24–26]. This problem needs massive public health advocacy about the importance and related risk of blood donation to ensure steady supply and availability of safe blood for transfusion.

## 5. Conclusion

In general, the study revealed that the proportion of adults who had adequate level of knowledge about blood donation and good attitude towards blood donation is high. However, the level of blood donation practice was low; and perception like not being fitted to donate blood, fear of being anemic after blood donation, fear of health risk after donation, and lack of information on where, when, and how to donate blood were the major reason for not donating blood. Educational status remained to be significantly associated with knowledge about blood donation. Regarding factors affecting attitude towards blood donation, religion was the only variable which remained to be significantly associated with attitude. Besides age, sex, religion, and self-perceived health status were statistically significant variables that affect blood donation practice.

## Additional Points

*Limitations*. The limitations of this study are similar to most of studies done on knowledge, attitudes and practices. One of the inherent limitations of such type of studies is that responses might be influenced by socially desirable traits and there might be the possibility of both interviewer and recall bias. The other limitation of this study is that the result cannot be inferred to other populations in the country because in multicultural countries knowledge, attitude, and practices regarding blood donation might be greatly influenced by tradition and sociodemographic factors of the population in different parts of the country.

*Recommendations*. National blood bank agency, district blood banks, WHO, and other organizations working on assuring safe and adequate blood supply should design strategies and tailored programs that promote blood donation practice. Besides, large scale in-depth behavioral studies need to be conducted to explore the distal and proximal societal factors that affect the communities' perception towards blood donation and blood donation practice.

## Figures and Tables

**Table 1:** Sociodemographic characteristics of adult population living in Gondar town, 2015 (*n* = 768).

Variables	Frequency (*n*)	Percentage (%)
*Age*		
20–25 years	402	52.3
26–30 years	232	30.2
31–35 years	79	10.3
36–40 years	55	7.2

*Sex*		
Male	430	56
Female	338	44

*Educational status*		
Below secondary school	225	29.3
Attend secondary school	189	24.6
Attend higher education	354	46.1

*Occupation *		
Student	194	25.3
Unemployed	166	21.8
Farmer	6	0.8
Daily laborer	28	3.6
Government employee	195	25.4
Own private work	165	21.5
Private employee	14	1.8

*Religion*		
Orthodox Christian	609	79.3
Muslim	101	13.2
Protestant Christian	46	6
Catholic and Jewish	12	1.5

*Marital status *		
Single	493	64.2
Married	210	27.3
Divorced	33	4.3
Widowed	16	2.1
Married but live in separated place	16	2.1

*Self-perceived health status*		
Excellent	206	26.8
Very good	231	30.1
Good	305	39.7
Poor	26	3.4

**Table 2:** Knowledge towards blood donation among adult population living in Gondar town, 2015.

Knowledge assessment items	Response	
	Correctly responded (*n* (%))	Incorrectly responded (*n* (%))
Place of blood donation	650 (84.6)	118 (15.4)
Importance of blood donation	678 (88.3)	90 (11.7)
Minimum age eligible for blood donation	338 (44)	430 (56)
Minimum weight eligible for blood donation	110 (14.3)	658 (85.7)
How often eligible individual can donate blood	259 (33.7)	509 (66.3)
Best blood donor type	654 (85.2)	114 (14.8)
Can pregnant women donate blood	524 (68.2)	244 (31.8)
Can women on menstruation donate blood	344 (44.8)	424 (55.2)
Can lactating women donate blood	366 (47.7)	402 (52.3)
Can diabetic patients donate blood	434 (56.5)	334 (43.5)
Can smokers donate blood	261 (34)	507 (66)
Maximum volume of blood being donated once	225 (29.3)	543 (70.7)

**Table 3:** Attitude towards blood donation among adult population living in Gondar town, 2015.

Attitude assessment items	Response	
	Correctly responded (*n* (%))	Incorrectly responded (*n* (%))
What do you think about blood donation	741 (96.5)	27 (3.5)
Do you think that donors will be exposed to infection during blood donation	511 (66.5)	257 (33.5)
Do you think donation is a moral duty	397 (51.7)	371 (48.3)
Do you think donation is harmful to donors	567 (73.8)	201 (26.2)
Do you think donation leads to anemia	486 (63.3)	282 (36.7)
Will you donate voluntarily for the future	608 (79.2)	160 (20.8)
Do you have a plan to donate voluntarily within the coming six months	356 (46.4)	412 (53.6)
Will you donate blood to an unknown person if you were asked	542 (70.6)	226 (29.4)
Will you ask for a monetary compensation for blood donation	719 (93.6)	49 (6.4)
Will you discuss blood donation with your friends and your family	599 (78)	169 (22)
Will you motivate others to donate	659 (85.8)	109 (14.2)

**Table 4:** Practice and frequency of blood donation and reason for donating and not donating blood among adult population in Gondar town, 2015.

Frequency and reasons	Blood donation practice	
	Ever donated (*n* (%))	Never donated (*n* (%))
Previous blood donation	141 (18.4)	627 (81.6)

How many times you donate		
One time	79 (56.0)	
2–5 times	59 (41.8)	
>5 times	3 (2.2)	

Reason for donation		
A friend or relative needed blood	55 (39.0)	
Voluntary	86 (61.0)	

Reason for not donating		
Fear of health problem		77 (12.3)
Fear of being anemic		79 (12.6)
Fear of weight loss		35 (5.6)
Since it is religiously prohibited		11 (1.7)
Since I have no time to donate		74 (11.8)
Since I have no information on when, where, and how to donate		106 (17.0)
I do not think I am fit to donate		133 (21.2)
Fear of needle		29 (4.6)
Since a friend/family told me not to donate		15 (2.4)
Since I do not like the idea of blood donation		55 (8.7)
Since I did not get the chance		13 (2.1)

**Table 5:** Logistic regression of knowledge towards blood donation with sociodemographic characteristics of adult population in Gondar town, 2015.

Variables	Knowledge status		Total	COR (95% CI)	AOR (95% CI)
	Adequate knowledge	Inadequate knowledge			
*Age*					
20–25 years	244 (60.7%)	158 (39.3%)	402	1.5 (1.06, 2.03)^$^	
26–30 years	119 (51.3%)	113 (48.7%)	232	1.00	
31–35 years	49 (62.0%)	30 (38.0%)	79	1.55 (0.92, 2.62)	
36–40 years	24 (43.6%)	31 (56.4%)	55	0.74 (0.41, 1.33)	

*Sex*					
Female	189 (55.9%)	149 (44.1%)	338	1.00	
Male	247 (57.4%)	183 (42.6%)	430	1.1 (0.8, 1.42)	

*Educational status*					
Below secondary school	88 (39.1%)	137 (60.9%)	225	1.00	
Attend secondary school	112 (59.3%)	77 (40.7%)	189	2.26 (1.53, 3.36)	2.28 (1.51, 3.44)^*∗*^
Attend higher education	236 (66.7%)	118 (33.3%)	354	3.11 (2.2, 4.41)	2.88 (2.01, 4.12)^*∗*^

*Occupation *					
Private employees	7 (50.0%)	7 (50.0%)	14	1.00	
Students	118 (60.8%)	76 (39.2%)	194	1.24 (0.42, 3.73)	
Unemployed	89 (45.9%)	105 (54.1%)	194	0.75 (0.25, 2.24)	
Farmers	0	6 (100%)	6	—	
Government employees	139 (71.3%)	56 (28.7%)	195	2.73 (0.9, 8.31)	
Own private work	83 (50.3%)	82 (49.7%)	165	1.04 (0.35, 3.14)	

*Religion*					
Orthodox Christian	348 (57.1%)	261 (42.9%)	609	1.00	
Muslim	52 (51.5%)	49 (48.5%)	101	0.8 (0.52, 1.21)	
Protestant Christian	27 (58.7%)	19 (41.3%)	46	1.1 (0.6, 1.96)	
Catholic and Jewish	9 (75.0%)	3 (25.0%)	12	2.25 (0.6, 8.4)	

*Marital status *					
Single	280 (56.8%)	213 (43.2%)	493	1.00	
Married	127 (60.5%)	83 (39.5%)	210	1.16 (0.84, 1.62)	
Divorced	12 (36.4%)	21 (63.6%)	33	0.44 (0.21, 0.9)^$^	
Widowed	6 (37.5%)	10 (62.5%)	16	0.46 (0.2, 1.28)	
Married but live in separated place	11 (68.8%)	5 (31.2%)	16	1.67 (0.57, 4.9)	

*Self-perceived health status*					
Excellent	130 (63.1%)	76 (36.9%)	206	1.61 (1.12, 2.31)^$^	
Very good	135 (58.4%)	96 (41.6%)	231	1.33 (0.94, 1.87)	
Good	157 (51.5%)	148 (48.5%)	305	1.00	
Poor	14 (53.8%)	12 (46.2%)	26	1.1 (0.49, 2.45)	

$ indicates significance in bivariate but not in multivariate logistic regression analysis, and *∗* indicates significant variable with *P* value less than 0.05 in multivariate logistic regression analysis.

**Table 6:** Logistic regression of attitude towards blood donation with sociodemographic characteristics of adult population in Gondar town, 2015.

Variables	Attitude		Total	COR (95% CI)	AOR (95% CI)
	Good	Poor			
*Age*					
36–40 years	41 (74.5%)	14 (25.5%)	55	1.00	
20–25 years	337 (83.8%)	65 (16.2%)	402	1.8 (0.91; 3.43)	
26–30 years	183 (78.9%)	49 (21.1%)	232	1.28 (0.64; 2.53)	
31–35 years	69 (87.3%)	10 (12.7%)	79	2.36 (0.96; 5.8)	

*Sex*					
Female	270 (79.9%)	68 (20.1%)	338	1.00	
Male	360 (46.9%)	70 (9.1%)	430	1.3 (0.9; 1.9)	

*Educational status*					
Below secondary school	176 (78.2%)	49 (21.8%)	225	1.00	
Attend secondary school	159 (84.1%)	30 (15.9%)	189	1.5 (0.9; 2.44)	
Attend higher education	295 (83.3%)	59 (16.7%)	354	1.4 (0.91; 2.12)	

*Occupation *					
Private employees	10 (71.4%)	4 (28.6%)	14		
Students	160 (82.5%)	34 (17.5%)	194	1.90 (0.56; 6.36)	
Unemployed	155 (79.9%)	39 (20.1%)	194	1.6 (0.5; 5.34)	
Farmers	4 (66.7%)	2 (33.3%)	6	0.8 (0.1; 6.25)	
Government employees	163 (83.6%)	32 (16.4%)	195	2.04 (0.60; 6.9)	
Own private work	138 (83.6%)	27 (16.4%)	165	2.04 (0.6; 7.0)	

*Religion*					
Orthodox Christian	498 (81.8%)	111 (18.2%)	609	1.00	1.00
Muslim	84 (83.2%)	17 (16.2%)	101	1.1 (0.63, 1.92)	1.1 (0.63, 1.92)
Protestant Christian	43 (93.5%)	3 (5.6%)	46	3.2 (0.97, 10.5)	3.2 (0.97, 10.5)
Catholic and Jewish	5 (41.7%)	7 (58.3%)	12	0.16 (0.05, 0.51)	0.16 (0.05, 0.51)^*∗*^

*Marital status *					
Single	408 (82.8%)	85 (17.2%)	493	1.00	
Married	174 (82.9%)	36 (17.1%)	210	1.6 (0.51, 5.1)	
Divorced	26 (78.8%)	7 (21.2%)	33	1.6 (0.49, 5.28)	
Widowed	10 (62.5%)	6 (37.5%)	16	1.2 (0.3, 5.1)	
Married but live in separated place	12 (75.0%)	4 (25.0%)	16	0.6 (0.12, 2.54)	

*Self-perceived health status*					
Excellent	175 (85.0%)	31 (15.0%)	206	3.0 (1.22, 7.31)^$^	
Very good	187 (81.0%)	44 (19.0%)	231	2.25 (0.941, 5.4)	
Good	251 (82.3%)	54 (17.7%)	305	2.46 (1.04, 5.81)^$^	
Poor	17 (65.4%)	9 (34.6%)	26	1.00	

$ indicates significance in bivariate but not in multivariate logistic regression analysis, and *∗* indicates significant variable with *P* value less than 0.05 in multivariate logistic regression analysis.

**Table 7:** Logistic regression of blood donation practice with sociodemographic characteristics of adult population in Gondar town, 2015.

Variables	Blood donation practice		Total	COR (95% CI)	AOR (95% CI)
	Ever donated	Never donated			
*Age *					
20–25 years	60 (14.9%)	342 (85.1%)	402	1.00	
26–30 years	35 (16.1%)	197 (84.9%)	232	1.01 (0.64; 1.6)	1.02 (0.64; 1.63)
31–35 years	26 (32.9%)	53 (67.1%)	79	2.8 (1.62; 4.82)	2.62 (1.6; 4.86)^*∗*^
36–40 years	20 (36.4%)	35 (63.6%)	55	3.26 (1.76; 6.02)	3.8 (2.0; 7.31)^*∗*^

*Sex*					
Female	47 (13.9%)	291 (86.1%)	338	1.00	
Male	94 (21.9%)	336 (78.1%)	430	1.732 (1.2; 2.54)	1.7 (1.14; 2.54)^*∗*^

*Educational status*					
Below secondary school	34 (15.1%)	191 (84.9%)	225	1.00	
Attend secondary school	39 (20.6%)	150 (79.4%)	189	1.46 (0.9; 2.43)	
Attend higher education	68 (19.2%)	286 (80.8%)	354	1.34 (0.85; 2.1)	

*Occupation *					
Private employee	3 (21.4%)	11 (78.6%)	14	1.00	
Student	30 (15.5%)	164 (84.5%)	194	0.67 (0.2; 2.55)	
Unemployed	27 (13.9%)	167 (86.1%)	194	1.6 (0.16; 2.26)	
Farmer	0	6 (100.0%)	6	—	
Government employee	57 (29.2%)	138 (70.8%)	195	1.51 (0.41; 5.63)	
Own private work	24 (14.5%)	141 (85.5%)	165	0.62 (0.16; 2.4)	

*Religion*					
Orthodox Christian	110 (18.1%)	499 (81.9%)	609	1.00	
Muslim	13 (12.9%)	88 (87.1%)	101	0.7 (0.36; 1.24)	0.63 (0.33; 1.2)
Protestant Christian	17 (37.0%)	29 (63.0%)	46	2.66 (1.41; 5.01)	2.62 (1.36; 5.1)^*∗*^
Catholic and Jewish	1 (8.3%)	11 (91.7%)	12	0.65 (0.1; 5.32)	0.51 (0.06; 4.45)

*Marital status *					
Single	72 (14.6%)	421 (85.4%)	493	1.00	
Married	51 (24.3%)	159 (75.7%)	210	1.9 (1.25; 2.8)^$^	
Divorced	7 (21.2%)	26 (78.8%)	33	1.6 (0.66; 3.76)	
Widowed	5 (31.2%)	11 (68.8%)	16	2.66 (0.9; 7.9)	
Married but live in separated place	6 (37.5%)	10 (62.5%)	16	3.51 (1.24; 9.95)^$^	

*Self-perceived health status*					
Good	40 (13.1%)	265 (86.9%)	305	1.00	
Excellent	49 (23.8%)	157 (76.2%)	206	2.1 (1.3; 3.28)	2.23 (1.4; 3.62)^*∗*^
Very good	44 (19.0%)	187 (81.0%)	231	1.56 (0.98; 2.5)	1.6 (0.97; 2.6)
Poor	8 (30.8%)	18 (69.2%)	26	2.94 (1.2; 7.22)	2.6 (0.99; 6.8)

$ indicates significance in bivariate but not in multivariate logistic regression analysis, and *∗* indicates significant variable with *P* value less than 0.05 in multivariate logistic regression analysis.
